# Selection, Optimization, and Compensation Strategies Used by Older Adults to Live Well With Technology: Qualitative Study

**DOI:** 10.2196/75019

**Published:** 2025-09-19

**Authors:** Wei Qi Koh, Kristiana Ludlow, Jacki Liddle, Nancy A Pachana

**Affiliations:** 1 School of Health and Rehabilitation Sciences, The University of Queensland, 84A Services Road, St Lucia, Brisbane, 4067, Australia, 61 734433773; 2 Centre for Health Services Research, Faculty of Health, Medicine and Behavioural Sciences, The University of Queensland, Brisbane, Australia; 3 Australian Frailty Network, The University of Queensland, Brisbane, Australia; 4 Occupational Therapy Department, Princess Alexandra Hospital, Brisbane, Australia; 5 School of Psychology, The University of Queensland, Brisbane, Australia

**Keywords:** technology, healthy aging, selective optimization and compensation, aging well, older adults

## Abstract

**Background:**

With rapid digitalization, technologies are increasingly integrated as part of our everyday lives and are becoming increasingly essential for individuals to participate in society. Technology presents opportunities to support healthy aging. Examples include digital health monitoring and opportunities to maintain social connectedness through online platforms. However, the processes in which older adults select and integrate technologies into their daily lives have not been well examined.

**Objectives:**

This study uses the Selection, Optimization, and Compensation (SOC) model to understand how older adults integrate technology into their everyday lives to live well. The two key research questions are as follows: (1) How do older adults describe their technology use and their choices, analyzed with respect to SOC processes? (2) How do older adults perceive that technology is a part of living well?

**Methods:**

A descriptive qualitative study was conducted. Purposive sampling was used to recruit older adults who were aged 55 years and older, were living in the community, spoke and understood English, and resided in Australia. Five in-person focus groups comprising 20 older adults were conducted. Data were analyzed using hybrid inductive and deductive reflexive thematic analyses, based on the SOC model.

**Results:**

All participants resided in Brisbane, Queensland. Older adults selected technology intentionally to enhance different aspects of their daily lives. Perceived “involuntary” selection of technology could lead to feelings of resentment or frustration. Optimization strategies included self-monitoring, integrating technology into daily routines, digital literacy and proficiency, and problem-solving skills. Compensatory strategies included choosing alternative technology that suited participants’ abilities or skills and seeking support through informal or formal avenues.

**Conclusions:**

These findings provide important considerations for technology developers to design technology in collaboration with older adults to ensure that they align with needs and preferences. Digital literacy is not sufficient to empower older adults to use technology; when empowering older adults to use technology, service providers should also consider facilitating other intrinsic and extrinsic resources and skills.

## Introduction

### Background

According to the World Health Organization, healthy aging entails creating the environments and opportunities that enable people to be and do what they do throughout their lives [1]. As populations are aging globally, healthy aging is a priority in many countries, and interventions and programs have been developed to support older adults to avoid negative impacts associated with disability or to maintain or improve their functional ability. Interventions have also focused on supporting older people to sustain or enhance participation in social engagement and other meaningful activities [2]. Examples include education, problem-solving and goal setting, leisure exploration [2], and activities to improve physical capacities [3]. Synthesized evidence suggests that physical activity programs can improve healthy aging by reducing physical limitations and improving psychosocial outcomes [4]. Similarly, non–exercise-based interventions, such as leisure-based interventions or programs to increase older adults’ self-awareness or self-acceptance, have led to improvements in older adults’ quality of life [2].

With rapid digitalization, technologies are increasingly integrated as part of our everyday lives and are becoming increasingly essential for individuals to participate in society. For instance, payments for services and goods have become digitized through mainstream telephone-based services, app-based services, and automated teller machines (ATMs) [5]. Correspondingly, technology presents new opportunities to digitize interventions aimed at supporting healthy aging [6]. Examples of technology that have been developed and leveraged to support healthy aging include web platforms, mobile health apps, and wearable technology, which have been designed to support older adults in self-monitoring and decision-making, measuring physiological, cognitive, or physical metrics, or delivering interventions [7]. While these technologies are intended to simplify and enhance older adults’ daily lives, they can also make everyday lives more complex [5]. To understand how technologies can be best used to support healthy aging, it is necessary to understand how they have been assimilated in older adults’ everyday lives. Several studies have been conducted to understand older adults’ experiences with technologies. For instance, Reyes and colleagues conducted a study in Australia to understand older adults’ choices of, and motivations to use technologies for health-related self-management [8]. They also sought to understand barriers and facilitators to technology use. The researchers found that older people leveraged technology for health monitoring and to remain active and connected. Facilitators include social and organizational influences, accessibility, and cost, whereas some barriers include perceived information overload, ease of use, and concerns about privacy and accuracy. These findings were echoed in other international studies conducted in Canada [9] and the United Kingdom [10]. While existing research has provided valuable collective insights into how older adults experience technology and barriers and facilitators to their use, the processes in which older adults select and integrate technologies into their daily lives have not been well examined.

### Selective Optimization With Compensation

The Selective Optimization with Compensation (SOC) model [11] is a meta-theoretical model focusing on the “how” of aging well; it describes processes that older adults undertake to reach their goals, particularly considering that their resources may become more limited over time. According to the SOC, when personal and environmental resources become limited, individuals make decisions to allocate these resources to pursue and to achieve their goals. This involves (1) selection, (2) optimization, and (3) compensation. Selection entails setting or prioritizing goals through loss-based selection or elective selection. For instance, older adults might choose to cease participation in certain activities that might become more effortful and instead select other activities deemed to be more meaningful. Optimization entails acquiring and refining resources and skills. Examples include learning new skills or knowledge needed to engage in chosen activities. Finally, compensation involves acquiring or substituting resources to achieve their goals. For example, older adults might leverage technology or support services to help them participate in activities. According to Freund and Baltes, the orchestrated use of these 3 action strategies effectively manages the loss of resources across individuals’ lifespans and conserves resources [12]. Given this, the SOC model could provide important theoretical guidance on bridging an established theory on successful aging with digital engagement. There is limited research on the application of the SOC model to the understanding of how technology is leveraged as part of, or integrated into, older adults’ process of selection, optimization, and compensation. This study aims to understand how technology is integrated into older adults’ everyday lives to live well using the SOC model. Specifically, the key research questions are as follows:

1. How do older adults describe their technology use and their choices, analyzed with respect to SOC processes?
2. How do older adults perceive that technology is a part of living well?

## Methods

A qualitative descriptive approach [13,14] underpinned our qualitative inquiry, where we stayed close to the “surface of the data and events” to gather rich descriptions of the views of participants and describe the phenomena from the viewpoints of participants.

### Sampling, Recruitment, and Context

Purposive sampling was used to identify and recruit participants who met the following criteria: (1) aged 55 years and older, (2) living independently in the community, (3) able to speak and understand English, and (4) residing in Australia. Different avenues were contacted to advertise this study to prospective participants. These included national and local senior networks, age-friendly university networks, and the University of Queensland’s (UQ) Healthy Living, an interprofessional clinic promoting healthy aging and well-being. Individuals who were interested in participating in this study were invited to share their preferred contact information with the research team. The lead researcher (WQK) contacted potential participants by email and/or a phone call to provide more information about the study and to arrange a focus group session at their preferred time and date. Online and in-person focus group options were planned, and recruitment was not limited to specific states in Australia. However, all participants who volunteered to join this study lived in Brisbane, and all focus group sessions were conducted in-person. No prospective participants declined participation. Brisbane is the capital city in the state of Queensland in Australia, with older adults constituting approximately 17% of the population. The local government, Brisbane City Council (BCC), has several initiatives to support seniors in Brisbane, and these include centers with organized activities, exercise programs, and transport concessions [15]. Brisbane has good digital infrastructure, and efforts have been made to improve digital accessibility and close the digital divide [16].

### Data Collection

Data collection took place between April and June 2024. Five face-to-face focus groups, comprising 20 participants in total, were conducted at a community health service for older adults. Each focus group comprised 3–5 participants and lasted approximately an hour. The topic guide (Multimedia Appendix 1) was developed and discussed with a consumer advisor. Each focus group was facilitated by a moderator (WQK). At the outset of each session, the moderator reiterated the research purpose, explained the session structure, and answered any questions. All participants were offered a AU $20 (US $14) gift voucher as a token of appreciation for their time and participation. The average duration of focus groups was 67 minutes. All sessions were audio-recorded and transcribed verbatim.

### Data Analysis

Data were imported into NVivo14 for data management. Data were analyzed using reflexive thematic analysis [17] both inductively and deductively based on SOC to identify strategies used by participants. First, WQK immersed herself in the data by reading and re-reading the transcripts and listening to the audio recording. During this process, data were coded descriptively and inductively through open coding. Next, codes were organized based on the SOC model and grouped into themes and subthemes. This was an iterative and reflective process, where the researcher moved back and forth between preliminary themes. Two meetings were arranged with the research team to discuss and explore the wider team’s perspectives (JL, KL, and NP) on the data and codes. For example, after organizing codes based on the SOC model, team discussions provided a sounding board as to whether data fitted into the preliminary themes. From discussions, subthemes like “passive selection of technology” were created to avoid “force-fitting” data into the “selection” component of the existing SOC model, acknowledging that while participants “select” technology, this could be based solely on circumstantial reasons. This process provided valuable leverage of multidisciplinary expertise to achieve a richer and more holistic interpretation of the data and the interrelationships between themes.

### Researcher Reflexivity

Considering the reflexive nature of our analytical approach, it is imperative to reflect on how the authors’ background and experiences shaped data analysis and interpretation [17,18]. The interviewer (WQK) is a lecturer in occupational therapy and an experienced qualitative interviewer, with more than 7 years of experience working clinically with older adults. This allowed WQK to understand and embrace older adults’ perspectives and drive to remain independent. As an international researcher from Singapore, WQK could miss out on participants’ nuances relating to local contexts in Australia. Field notes were taken and discussed with the research team (JL, KL, and NP) who contributed their professional and research expertise during data analysis to situate our findings in both local and international contexts. They also contributed their expertise in situating findings with theoretical underpinning.

### Trustworthiness

This study followed a methodologically rigorous process, based on Lincoln and Guba’s criteria for trustworthiness [19]. First, to ensure transparency and confirmability, data management and analysis of the reflexive thematic analysis process took place on the NVivo software. Next, findings were discussed with the research team during the analysis process to obtain a more holistic, multidisciplinary perspective of the data collected, providing credibility. Another strength is in its use of an established theoretical framework (SOC) to guide data analysis. In addition, the criterion of transferability was aided through having clear descriptions of participants’ characteristics and direct quotations. The Standards for Reporting Qualitative Research (SRQR) checklist was to report the conduct of this study (Checklist 1) [20].

### Ethical Considerations

Ethical approval was obtained from the University of Queensland (Ref:2023/HE002389). Written informed consent was obtained from all participants, who were also provided with the opportunity to clarify questions prior to the commencement of data collection. Participants' data were stored securely and confidentially in alignment with approved institutional ethical guidelines. Each participant was provided a AU $20 (US $14) gift card as a token of appreciation for their time for participation. To ensure anonymity, participants were given pseudonyms for data analysis and reporting.

## Results

### Demographic Characteristics

Participants’ demographic information is summarized in Table 1. Participants’ mean age was 75.4 years, and there was a similar proportion of men and women. More than half of the sample (n=12, 60%) had a Bachelor’s degree or higher, and most participants were unemployed or retired. All participants (n=20, 100%) were regular users of smartphones, computers, or laptops. Most described proficiency and daily use of these technologies. Approximately two out of three have had experiences using pedometers or smartwatches; one third are regular users (n=6, 30%) and others have seen or used them previously (n=7, 35%). None of the participants had used virtual reality technologies. Strategies relating to technology use will be described using the SOC model with 3 overarching themes: (1) elective selection of technology— which are further divided into 2 subthemes: “elective, active selection” and “passive selection”; (2) optimization strategies, and (3) compensation strategies.

**Table 1.** Participants’ characteristics.

Characteristics	Participants
Gender, n (%)	
Men	11 (55)
Women	9 (45)
Mean (SD) age, years (range)	75.4 (68-82)
Educational attainment*,* n (%)	
Bachelor’s degree	12 (60)
Diploma/advanced diploma	7 (35)
Certificate III/IV (vocational qualification)	1 (5)
Employment status, n (%)	
Part-time or casual employment	2 (10)
Retired	18 (90)

### Theme 1: Elective Selection of Technology

This theme described ways in which participants intentionally and unintentionally chose to use technology to facilitate different aspects of participation in daily living and well-being. The subthemes include (1) elective, “active” selection of technology and (2) passive selection of technology, where participants chose technology but perceived the choice as being “forced” or unavoidable.

### Subtheme 1—Elective, “Active” Selection of Technology

All participants described regular use of their mobile phones, laptops, or personal computers, and a few used other technologies, such as iPads, tablets, and smartwatches. Participants conveyed that technologies and applications were purposively selected to help with daily life organization. For example, some described keeping digital records of their accounts or finances, organizing photographs or photobooks electronically, or moving from using physical diaries or calendars to digital versions for more effective management of schedules and appointments.

I never seem to have the calendar ... besides, I share it with my husband, so he can see what it’s like. When you get older, as so many… medical, specialists… we’re trying to keep track of it all.

[Olivia, 74]

Some participants described using technology as a means for leisure and social participation. For instance, a few shared their experiences engaging in mobile games such as quizzes or crosswords, which were described as fun or as means to pass time. Technology was said to provide opportunities for mental stimulation and lifelong learning through information seeking and learning through the internet. Examples included tracing family history and increasing knowledge in various topics of interest, such as medications, artificial intelligence (AI), or other educational topics. Participants expressed that technology provided opportunities to feel connected with the world:

Yeah. I’m a great believer in current affairs. If I have a few moments to scroll, I’m on Twitter … the commentary on things that are going on is amazing. You just feel so in touch with the world, you know?.

[Liam, 69]

A few participants described using activity tracking applications to monitor daily activities, such as physical activity levels (eg, step count) and physiological features (eg, heart rate and sleep). One participant expressed that this served as supplementary information that was provided to his general practitioner (GP) during medical consults:

It’s handy to have, particularly in a medical interaction, like I go to my GP, and I’ve been recently regularly, two times a month perhaps, for a variety of different reasons, and I like to be able to say that this is what I’m doing, this is how I’m going, and she can monitor my overall activity rates and things like that … I find that particularly useful.

[John, 72]

In addition, participants reported using technology to stay socially connected with family and friends, particularly those living overseas; to reconnect with old friends; and to connect with new friends and likeminded people:

I use the cliche apps like Facebook to keep up with friends back in Hungary, family in Australia … Fair few discussion groups on various things like cooking.

[Liam, 69]

… maintain old friendships. Yeah. And renew old friendships … I didn’t know that [name of friend] would age so well, oh that’s great, I’m going to the hairdressers (laughs).

[Hazel, 69]

### Subtheme 2—Passive Selection of Technology

Many participants described technology uptake as passive or “forced,” where technology selection was seen as essential or unavoidable, regardless of their willingness to use them. This was described as resulting from rapid digitization of services such as banking and electronic commerce. Participants conveyed that this could affect or threaten older adults’ participation in meaningful activities and daily routines:

We’re forced into technology. The thing is you haven’t got any choice anymore because essentially when I had to get the blue card for PCYC [Police Citizens Youth Club – a network of non-profit organisations], the only way you could do it was online… the other thing is that I just got my license renewed and I thought, well, I’ll see if I can get this new digital license. The only way you can get your digital license is you’ve got to download an app.

[Jack, 76]

While most participants identified themselves as being relatively proficient in navigating technology, feelings of being forced into using technology in their day-to-day lives were expressed and described as frustrating and upsetting. They explained that this affected how they engage in activities:

I’m annoyed and angry with technology… for instance, now… you’re running a book sale, but you’ve got to register it, and if you want to register it, you’ve got to have an account, and you’ve got to have a code name, a code word, and you’ve got to nominate a time. I’ve got friends up at [regional book club], people who love books, but they refuse to sign up because they can’t nominate a time to get there from [regional area] by public transport ….

[Samuel, 80]

### Theme 2: Optimization Strategies

This theme described strategies that were identified by participants to optimize the use of their selected technology to ensure meaningful life participation. It also described how participants optimized their life using technology. These included one’s ability to self-monitor, to successfully adjust to and/or integrate technology to fit into daily lives, and to leverage one’s digital literacy, positive attitude, and problem-solving skills.

Many described the importance of monitoring the relevance of technology, keeping in mind their intended use of technology for well-being. In other words, they described active deliberation about when technology can be, or has been, beneficial in helping them live well. Some participants expressed the importance of using technology purposefully to avoid time wastage or harmful effects:

I choose what I like to see, who I want to talk, and also connecting with family. WhatsApp, I call my sister once a month or once a week. We talk about things. It’s something that I really like … not the silly thing of scrolling and scrolling and scrolling [social media], and spending hours doing … that young people do? No. I decide what to do.

[Evelyn, 76]

Participants explained that ongoing deliberation about the utility of technology encouraged them to continue to engage with their chosen technological mediums in some instances and disengage in other instances. For example, while some described leveraging mobile applications to track their daily activities (eg, step count) for self-monitoring, they reported eventually disengaging from these apps after gaining a sense of their activity levels to benefit from the activity without the drawback of the technology:

I found that helpful in the beginning, but I just don’t use it anymore. I have an idea of how long my walks are … I’d rather enjoy the walk then look … if you go out and, you know, bush or national parks or something, you want to look at your surroundings. You don’t want to be embedded in this thing [mobile phone].

[Amelia, 80]

Another optimization strategy was the investment in time and resources to keep updated with technology and be aware of potential risks, such as misinformation or financial scams. Some participants identified digital proficiency, previous professional experience, or personal experiences as assets that enable them to problem-solve through challenges. For example, to keep updated with technology updates, one participant actively acquires knowledge through searching for YouTube videos. A few participants also described the importance of having a positive attitude in seeking resources or in equipping oneself with skills to overcome current or prospective challenges, such as risk management or active problem-solving:

We’ll have problems, but we need the confidence to think, ‘Oh yeah, look, a little bit of sorting out.’ I mean, you know, we’ll be able to understand it, ask. Whereas some people are just so confused, it’s too much for them. But I think if you have a healthy attitude towards aging, you think, ‘Well look, I’ll make sense eventually, I’ll seek out help, I’ll look at the YouTube videos, I’ll work it out.

[Luke, 79]

### Theme 3: Navigating Technology Use and Compensation Strategies

This theme described challenges that participants encountered during technology use and strategies that were discussed as ways to adapt to challenges or limitations; these included choosing alternative technologies that are better aligned with their knowledge and abilities and seeking formal and/or informal support. These strategies enabled them to continue engaging with technologies in ways that support their lifestyles and routines without perceived detractions.

Some participants described functional changes or disabilities that limited their abilities to use technology effectively and safely. One participant provided the example of visual fatigue when looking at smaller fonts, particularly through her mobile phone. Correspondingly, she described choosing an alternative technology that was more suited to her physical abilities (eg, laptop with a larger font size). Some participants also described experiencing challenges or feeling stuck when they experienced technical issues or when they were not able to navigate technology use:

I was doing a post to Facebook a couple of days ago, and I wanted to reorder the photographs that I was taking, and they were in an event, and I wanted them to be sequential, and I thought, ‘Damn it, I don’t know how to do it. I want to get this post out; I don’t have time now.

[Luke, 79]

A few participants described seeking informal support from their friends or family members, particularly the younger generation whom they described as being more technology-savvy:

I must admit I call him [husband], as when I get stuck, he’s very good with technology. And I just call him ... if he fails, we call [a service] or whatever it is

[Emily, 71]

Or a 15-year-old grandchild

[Hazel, 69]

One participant expressed concerns that relying on family members to assist with technology could lead to burden:

My daughter occasionally drops in and tries to sort out things and explain things, but I don’t want to burden her anymore.

[Samuel, 80]

A few participants described seeking external support from other sources, such as a local nonprofit organization that assists people who are aged older than 50 years to become competent in computer and internet use:

I am going to shortly join a group that’s like a technical men’s shed [non-profit organization to promote socialization and shared activities among men] … I’m hoping my technological problems like reordering the photographs on Facebook and that sort of thing will be handy … to figure out these little perks that I don’t quite know at the moment yet.

[Luke, 79]

## Discussion

### Principal Findings

This research aimed to understand ways in which older adults perceive they use technology to live well. Specifically, this work utilized the SOC model to understand the “hows”; how older people select or opt out of using technology (selection), how they optimize their resources and skills to use technology to live well (optimization), and how they compensate for a lack of resources and skills (compensation) to maintain meaningful participation in the face of challenges. Corresponding with the *“*elective selection” ontology of the SOC model, findings revealed that older adults selected technology intentionally to enhance different aspects of their daily lives. Perceived “involuntary” selection of technology could lead to feelings of resentment or frustration. In some instances, this led to participation in activities or routines that did not involve technologies. Corresponding with the “optimization” component of the SOC model, optimization strategies where older adults used technology to improve daily living (and vice versa) included self-monitoring, technology integration into their daily routines, digital literacy and proficiency, and problem-solving skills. Corresponding with the “compensation” aspect of the SOC model, participants’ compensatory strategies included choosing alternative technology that suited participants’ abilities or skills and seeking support through informal or formal avenues. Our participant cohort was living in Brisbane, Queensland, with good digital infrastructure across the city; the cohort identified themselves as being relatively digitally savvy and well-educated. Given this, findings may be more resonant with this population.

### Technology Selection—The “Whats” and “Whys”

Participants in our study reported voluntarily and purposefully selecting technologies that facilitated improvements in various aspects of their daily lives, such as improving the effectiveness and efficiency of self-management (eg, activities within daily routines, health management) and participating in leisure, social, cognitive, and physical activities. These are congruent with the literature on how older adults perceive successful aging [21]. The most frequently used technologies were smartphones, laptops, and/or desktop computers, and the most commonly discussed technological applications were social media and messaging apps, internet searches, digital organizers, and calendars. Purposeful and meaningful selection of technology appeared to encourage uptake and continued use. Our work bolsters findings from several studies showing that the perceived benefits of technology and their meaningfulness facilitated uptake [22], whereas low interest or a lack of understanding about the purpose of technology impeded older adults’ use or continued use [23-25]. A possible explanation is that purposeful, autonomous selection of technologies empowers older adults with intrinsic motivation, which could in turn encourage sustainable use of technology beyond the novelty period [26]. On the contrary, some technologies were selected nonautonomously or, in some participants’ words, being “forced” upon them with circumstantial changes. These might have been further affected by the COVID-19 pandemic, which has led to a particular acceleration of service digitalization [27,28]. When technology use is perceived as involuntary, this could lead to reduced satisfaction, a sense of oppression, or frustration [29]. These findings highlight the importance of ensuring autonomy and providing older adults with a choice in selecting technologies that are seen as meaningful in improving their daily living. This is particularly important given the continual interest and development of technology to support older adults to live well.

### Technology Use—The ‘Hows’

Participants in this study described leveraging different cognitive skills, including metacognition and problem-solving skills, to optimize the use of their selected technology. Metacognition entails actively monitoring and evaluating information to ascertain whether one is moving toward a goal and making decisions on how to make changes to achieve the goal [30]. In our study, older adults described metacognitive processes used to ensure that they use their selected technology in ways that aligned with their personal preferences, lifestyles, and routines. Such monitoring and self-regulation skills have been found to improve health outcomes, such as preventing illness and maintaining health [31].

Older adults also described ways in which they overcame problems that were encountered, such as technical issues or working around software or hardware updates. Such problem-solving skills have been viewed as an important aspect of successful aging [32]. According to Chen and colleagues, problem-solving entails having “fluid resources,” which encompasses abstraction and performing mental operations, and crystallized resources, which entail gaining resources through cumulative experience and knowledge [32]. Given that fluid resources are based on reasoning and processing abilities, they can decline with age, particularly in later adulthood [33,34]. On the contrary, crystallized resources can be maintained or increased over time up to late adulthood [34]. Our participant cohort was generally highly educated. Most reported high digital literacy skills as part of their previous professional employment. This might have contributed to the development of crystallized resources, which allowed them to work through issues and to consider other avenues, such as YouTube tutorials, as a means to equip themselves with the skills and knowledge to overcome challenges. In addition, technology is rapidly developing alongside potential benefits and risks such as misinformation and online scams or fraud. According to National Seniors Australia, more than 600,000 scam reports were made in 2023 with older adults being most impacted [35]. Likewise, the National Council of Aging revealed that worldwide, older adults aged 60 years and older reported US$3.4 billion total fraud loss in 2023 [36]. Accordingly, problem-solving skills appear to be requisite to afford older adults the confidence and abilities to use technologies in calculated ways. This includes ensuring that the benefits of technology outweigh their risks and for technology use to be meaningfully aligned with older adults’ personal well-being goals.

Some participants described a positive outlook and a “can do” mentality as being an important strategy for technology use. Positive attitudes have been found to be a psychological resource in healthy aging [37,38] as studies have found that older people with a more positive attitude were more likely to have better self-reported health and well-being [39], participate in community and physical activities [40], and activate health-promoting behaviors [41]. In our study, such behaviors include trying to find solutions to overcome potential problems encountered to continue using technology in a meaningful way. This suggests that when designing interventions to support older adults to engage in technology, considerations should also be made to inculcate positive attitudes and consider measuring attitudinal changes as an outcome measure.

Some older adults may lack the confidence or ability to use technology in ways that they perceive as meaningful. For instance, some participants in this study expressed a lack of confidence to overcome issues encountered and may lack “crystallized resources” such as digital proficiency to navigate issues. In such instances, support appears crucial, including informal support from family members and formal support from organizations. While informal support is valuable, some study participants also expressed concerns about burdening their family, particularly if they sought help more frequently than they hoped. Such self-perceived burden can affect older adults’ self-concept [42]. Considering that technology is continuously evolving, it is more than likely that any users of technology, including older adults, may require support to troubleshoot potential issues. While family members might be able to bridge obstacles [43], it is important to consider sustainable ways, such as community training hubs, which older adults can receive technological support as required. While such services might be available, many participants in this study did not seem aware of such services; this resonates with findings from other studies [43,44].

### Implications for Practice

There is increasing interest in using technology to support healthy aging, as seen through continued development and research in this field, alongside trends of rapid development of digital infrastructure in developed countries worldwide. Despite this, a lack of technology uptake and sustained use remains a pertinent issue [25], and efforts continue to be made to improve digital uptake particularly among older adults nationally [45] and internationally [46]. Our findings provide practical implications to support aged care organizations, centers, and care providers to support older adults to use and integrate digital technologies into their daily lives. Regarding technology selection, our study suggests that meaningful selection of technology by older adults provides them with a sense of autonomy and motivation for sustained use. Selected technologies must serve a purpose, such as meaningfully supporting aspects of their daily lives. Moving forward, aged care organizations, centers for seniors, and health care professionals working to prescribe and equip older adults to use technologies should consider empowering them with the autonomy to reflect on their needs and abilities and to choose technologies that can be personalized to their routines and preferences [47]. Forced selection can be counterproductive in supporting older adults’ well-being, and it must be acknowledged that older adults may opt out of using technologies. If forced selection occurs, additional supports are likely necessary to support older adults in using technologies and to mitigate the risk of excluding participation.

In terms of optimization strategies to use technology purposefully, cognitive processes, including metacognition and problem-solving, appear necessary to empower older adults with the confidence to navigate the use of technology in ways that align with their intended purpose. This is particularly true of technologies that are dynamic, which can affect ways of using or operating them [48]. Likewise, a positive attitude toward using technology and its challenges appears to be an important, facilitatory resource. Therefore, service developers or health care professionals working to equip older adults with technological skills should also consider the cognitive aspects of using technology and empower older adults to gain skills and knowledge to use them in reasoned ways. Examples might include empowering older adults with problem-solving skills [49], building self-efficacy and confidence in managing prospective challenges, or positive thinking training [50]. Regarding compensatory strategies, being supported to use technologies and navigate challenges is important [51]. Such resources may include family support and external support services like community hubs. Given that not all older adults may have family support, it is important to ensure that external support services are readily accessible to promote digital inclusion [52] and to minimize the negative repercussions of digital exclusion, such as poorer health outcomes and social isolation [53].

In terms of leveraging the SOC model as a theoretical framework—our findings underlined its utility and success in providing an established framework to guide our analysis and interpretation of the “how” of individual older adults could integrate technologies into their daily lives. However, individuals’ goals and strategies are often interrelated with contextual demands. For example, our subtheme “passive selection of technology” was largely influenced by participants’ perceptions and experiences of a rapidly developing digital infrastructure that influenced their uptake of technology. However, the SOC model provides limited consideration of contextual and societal factors that can influence the aging process. Accordingly, researchers leveraging the SOC model to understand digital engagement should take into consideration its limitations and reflect how contextual demands influence individuals’ goals and strategies.

### Limitations

The limitations of this study must be acknowledged. While several recruitment avenues were used to recruit participants, all participants were recruited from a healthy aging clinic and were relatively physically active. There was no option in our demographic survey to capture data of frequency of technology use in a week, and this should be acknowledged as a limitation given that this knowledge could provide more information on participants’ digital proficiency. However, most identified and described themselves as being technologically savvy and relatively well-educated, with all reporting weekly use of computers/laptops and smartphones. Participants’ digital proficiency could lead to response bias, and findings from this research may be more resonant with this population and may be less representative considering other demographic groups, particularly older adults who are less active and technologically savvy. Finally, this study was limited to participants who could speak English, and as such, our findings may have excluded non-English speaking older adults’ perspectives and experiences. Future studies might consider further exploring understanding technology use and non-use in other population groups.

### Conclusions

Framed with respect to the SOC model, this study contributes to a theoretical understanding of how older adults use technology to live well. Older adults purposefully select technologies that align with their priorities in living well, and this appears to be a facilitator for technology uptake and sustained use. Older people leverage different resources and skills to use their selected technologies in meaningful ways. These include metacognitive skills, problem-solving skills, positive attitudes, and internal and external support. These findings provide important considerations for technology developers to design technology in collaboration with older adults to ensure that they align with needs and preferences. It is also evident that digital literacy is not sufficient to empower older adults to use technology; when empowering older adults to use technology, service providers should also consider facilitating other resources and skills.

## Supplementary material

10.2196/75019 Multimedia Appendix 1 Topic Guide.

10.2196/75019 Checklist 1 Standards for Reporting Qualitative Research (SRQR) Checklist.

## Abbreviations

AI: artificial intelligence
BCC: Brisbane City Council
SOC: selective optimization with compensation
