# Supplementary material for: Selection, Optimization, and Compensation Strategies Used by Older Adults to Live Well With Technology: Qualitative Study
Source: JMIR Aging. 2025 Sep 19;8:e75019. doi: 10.2196/75019 (PMC12448253; doi:10.2196/75019)
Supplement: Multimedia Appendix 1 [file aging-v8-e75019-s001.docx]

**Supplementary Material 1: Topic Guide**

1. **What technology are you using (in general)?**

Provide examples of technology prompts

- - Equipment: e.g., mobile phones, computers, tablets, smart watches
  - Technology mediums: Apps, podcasts, websites (e.g., YouTube, social media)

1. **How do you think this technology improve(s) your day-to-day life?**

- How do you think technology can better help you…
  - stay connected socially with your friends, family or meet new friends?
  - keep a healthier lifestyle?
  - keep mentally active?
  - stay connected with your community?
  - or anything else that you can think of?

1. **Have you faced any challenges when using this technology?**(Refer back to the technology that was/were discussed earlier)

- What has been difficult?
